# Differences in fish herbivory among tropical and temperate seaweeds and annual patterns in kelp consumption influence the tropicalisation of temperate reefs

**DOI:** 10.1038/s41598-022-24666-9

**Published:** 2022-12-08

**Authors:** Adriana Vergés, Brendan S. Lanham, Madoka Kono, Satoru Okumura, Yohei Nakamura

**Affiliations:** 1 Centre for Marine Science and Innovation, School of Biological, Earth and Environmental Sciences, University of New South Wales, Sydney, NSW 2052 Australia; 2 Sydney Institute of Marine Science, Mosman, NSW 2088 Australia; 3 National Centre for Coasts and Climate, University of Melbourne, Parkville, VIC 3052 Australia; 4 Graduate School of Integrated Arts and Sciences, Kochi University, Monobe 200, Nankoku, Kochi Japan; 5 Faculty of Agriculture, Kochi University, Monobe 200, Nankoku, Kochi Japan

**Keywords:** Climate-change ecology, Marine biology, Ecology, Climate-change ecology, Community ecology

## Abstract

Climate change is leading to novel species interactions and profoundly altering ecosystems. In marine systems, tropical and subtropical species are increasing in higher latitudes. This has been linked to the deforestation of temperate coastlines, as direct effects of ocean warming combine with increased herbivory from tropical and sub-tropical fishes and lead to the decline of canopy-forming kelp. Here, we tested the hypothesis that this deforestation may be facilitated by greater palatability of temperate kelp and other canopy seaweeds compared to tropical taxa. We used multiple-choice filmed feeding field experiments and chemical analyses to measure the palatability of temperate and tropical seaweeds from Tosa Bay (southeastern Japan) and we used single-species feeding assays to measure changes in consumption of the kelp *Ecklonia cava* throughout the year. We found no evidence that temperate seaweeds are more palatable to herbivorous fish. In the multiple-choice assays, consumption was concentrated on both tropical and temperate *Sargassum* species, which are ephemeral and peak in abundance in the spring/early summer. Consumption of the kelp *Ecklonia cava* peaked during the autumn, when *Sargassum* species are absent. The highest levels of kelp herbivory coincide with the reproductive season for *E. cava* and may contribute to the long-term decline of these kelp forests in southern Japan.

## Introduction

Climate change is shifting the distribution of species^1^ and altering ecological assemblages, as not all taxa respond to warming by shifting their range, and species that move do so at different rates^2^. This reorganization of ecological communities can lead to some biotic interactions being lost, when interacting species no longer co-occur in space, and can also lead to new interactions among previously separated taxa^3^. In some instances, these climatic impacts on species interactions can outweigh or even reverse the direct effects of climate^4,5^.

Climate-mediated disruptions in biotic interactions linked to species redistributions are already impacting the health of ecosystems and humans, as well as our economy^6^. Documented impacts range from increased impacts of pests and diseases to unpredictable fisheries and crop yields^6,7^. A better mechanistic understanding of how climate change alters species interactions is therefore key to understand and predict future ecosystem function.

Changes in species assemblages are manifesting at particularly rapid rates in marine ecosystems^1^. Species redistributions are causing a global ‘tropicalisation’ of marine communities, as species from the tropics become increasingly abundant in higher latitudes^8^. Plant–herbivore interactions are particularly important in the ocean^9^ and have been significantly impacted by climate change and tropicalisation^10,11^. Around the world, range-expanding tropical and subtropical herbivores are overgrazing canopy seaweeds and removing their recruits, causing regime shifts towards systems dominated by low biomass turf algae or urchin barrens^8,10,12^. In particular, in recent years the range expansion of tropical and warm-temperate herbivorous fish has been linked to the deforestation of seaweed forests around the world, including in Japan^13^, the eastern Mediterranean^12^ and the eastern and western coastlines of Australia^10,11^.

The deforestation of temperate reefs by range-shifting herbivorous fishes may be facilitated by functional differences in seaweed-herbivore interactions between tropical and temperate systems^8,14^. In particular, herbivorous fishes in the tropics are much more diverse taxonomically and functionally than in temperate reefs^15^. The tropics are also characterized by higher rates of fish herbivory than temperate systems^16^. These patterns have been linked to differences in the palatability of seaweeds, with some evidence that tropical seaweeds may be less palatable and better defended chemically than temperate taxa^17^. Further, nitrogen content, a critically important nutrient for herbivores, is generally higher in temperate plants and macrophytes than in tropical species^18^ and may therefore further stimulate consumption. These patterns have led scientists to hypothesize that the range expansion of tropical herbivorous fish into higher latitude reefs may be facilitated by the presence of more nitrogen-rich and weakly defended seaweeds in temperate reefs^14,19^.

However, most mechanistic palatability assays focusing on identifying specific nutritional traits that mediate feeding have focused on invertebrates^14,17^ which are functionally different from fish in terms of their mobility and their ability to produce the cellulase necessary to digest brown algae^20^. Gaining a better understanding of how the palatability of brown seaweeds varies among species and what factors influence fish herbivory are particularly important in the context of continued range expansions by tropical herbivorous fish, as these seaweeds include the most important habitat-formers in temperate reefs (e.g., Laminariales and Fucales) and fish are the taxa that are shifting their distribution polewards at the fastest rate^21^.

Here, we experimentally test the hypothesis that temperate brown seaweeds are preferentially consumed by fish in a tropicalised coastline because they are more nutritious and less chemically defended than tropical brown seaweeds. The palatability of co-occurring temperate and tropical seaweeds was measured in the field in southeastern Japan. This region is a global hotspot for environmental change in response to ocean warming where tropical fishes are now dominant^22^. Temperate kelp forests have been decreasing in these reefs since the late 1990s^23,24^, while coral cover and diversity have increased extensively^25,26^. Further, a rapid shift from temperate *Sargassum* species (e.g., *S. micracanthum* and *S. yamamotoi*) to tropical *Sargassum* species (e.g., *S. ilicifolium* and *S. carpophyllum*) has been occurring since the late 1980s^23^. Although the direct effect of rising water temperatures on seaweed physiology is considered an important factor for long-term changes in seaweed vegetation in this region (seaweed bed decline and algal species changes)^23,27^, it is still unclear how indirect effects such as increases in fish herbivory may be involved in these changes. Serisawa et al.^27^ reported fish damage on kelp blades and bladelets in Tosa Bay. Further, herbivorous fish damage to canopy-forming seaweed (Laminariales and Fucales) populations has also been reported from several regions in Japan since the late 1990s^28–32^. Since similar coastal environment changes and increases in herbivorous fish abundance/activity are also occurring in other temperate locations of the world^11,33^, assessing whether and how herbivorous fish preferentially consume temperate seaweeds can contribute to understanding the potential mechanisms facilitating the decline of kelp and the range expansion of tropical seaweed species.

To assess whether herbivorous fish are selectively feeding on temperate seaweeds and whether the physiology and nutritional value of seaweeds are related to this feeding preference, we used a combination of multiple-choice filmed feeding experiments and chemical analyses of co-occurring temperate and tropical canopy-forming brown seaweeds. This included the dominant kelp in the study region, *Ecklonia cava*, a perennial species characterized by high biomass throughout the year, as well as dominant temperate and tropical *Sargassum* species, which are ephemeral and have relatively short periods of high vegetation cover during the winter–early summer^34^. The feeding experiments were performed during summer, and subsequent assays with the kelp *Ecklonia cava* (the only canopy species present year-round in the region) were performed on a monthly basis to characterize changes in kelp consumption throughout the year^35^.

The specific questions we asked are: (i) Are herbivorous fish abundance and biomass higher at tropicalized locations than at kelp-remained locations? (ii) Are temperate brown seaweeds consumed preferentially by herbivorous fishes? (iii) Are fish feeding preferences mediated by seaweed nutritional or chemical defence traits? (iv) How does consumption of the dominant perennial kelp *Ecklonia cava* vary temporally?.

## Materials and methods

### Study locations and study species

This study was performed in Tosa Bay in Kochi prefecture, southeastern Japan (33°N, 133°E). Fish and benthic surveys were performed at two coral-dominated (Tei and Yokonami) and two nearby kelp-dominated locations (Usa and Tanoura; Fig. S1). Usa and Tanoura are the only remaining kelp forests in the region. Tei and Yokonami are known as tropicalised locations. Extensive kelp beds (180 ha) existed at Tei^23^, but corals have rapidly developed since the disappearance of kelp in the early 2000s^8^. At Yokonami, *Sargassum* beds were present until the 1980s, but corals have now developed^22^. In situ feeding experiments were performed at the coral dominated Tei and Yokonami only, where herbivorous fish biomass was higher (see Results). Surveys were performed in 2014 during summer (July), which represents the biomass peak of all *Sargassum* species^34^. *Sargassum* species are ephemeral and disappear from mid summer onwards^34^. An additional bioassay experiment was set up in October 2014 to measure consumption rates of the perennial kelp species *Ecklonia cava* in the absence of *Sargassum* species. This was followed by monthly bioassay deployments as described below. The water temperature in central Tosa Bay varies seasonally between 15–16 °C (February) and 28–29 °C (August)^22,34^.

We used six canopy-forming brown algae in our feeding experiments, three of tropical distribution (*Sargassum ilicifolium*, *S. carpophyllum*, and *S. alternato-pinnatum*) and three of temperate distribution (*Ecklonia cava*, *S. micracanthum,* and *S. patens*). All species are abundant canopy-forming algae in Tosa Bay^23^. Their depth distribution is between 1 and 10 m. Seaweed palatability may vary between populations in regional and latitudinal scales^17^ probably because seawater nutrient conditions may affect palatability^36^. This study aimed to compare the differences in palatability among macroalgae species therefore all seaweeds were collected from the same location (Pacific Ocean side of the Yokonami Peninsula, central Tosa Bay). Although the epiphytes are considered important food resources for browsing parrotfishes^37^, we did not remove epiphytes in this study because (1) we targeted not only parrotfish but also all herbivorous fishes, and (2) we aimed to clarify the selectivity of herbivorous fishes for each macroalga species under natural conditions. We avoided using macroalgae with high epiphytic load in the experiments. The experimental seaweeds were collected on the day or a day before the experiment. The latter were maintained in a large, free-flowing seawater tank (2 t tank at the Usa Marine Biological Institute, Kochi University) until the experiment day and then transported to the experimental sites using lidded buckets with aeration under appropriate cool condition.

All methods were carried out in accordance with the UNSW Animal Research Ethics Procedure. An Animal Ethics permit was not necessary in this instance as fish were filmed in their natural environment and there was no direct interaction or manipulation of fish.

### Characterisation of herbivorous fish and benthic communities

The herbivorous fish and benthic community composition of each reef were quantified in July 2014 using n = 5 transects haphazardly deployed between 9 am and 3 pm and separated by at least 10 m. Herbivorous fish assemblages were surveyed by the same expert (Yohei Nakamura) using underwater visual surveys (25 m long, 5 m wide) by identifying all individual fish to species. Total length of each fish was estimated to the nearest cm (roving fishes) and 0.5 cm (resident fishes). Length estimates of individual fish were converted to biomass using the allometric length–weight conversion W = *a* * TL^*b*^, where W = weight in grams, TL = total length and parameters a and b are constants obtained from Fishbase (www.fishbase.se). The total abundance of herbivores per transect was contrasted among habitat type (coral and kelp; fixed factors) and location (coral: Tei, Yokonami; kelp: Usa, and Tanoura; fixed factors nested in habitat—these were considered fixed as the kelp sites represent the only kelp-dominated sites in the region) using a negative binomial generalised linear model. The negative binomial distribution was used as it provided the best data fit. The total biomass of herbivorous fish per transect (log transformed) was contrasted among the same covariates using a linear model. Models were run using the manyglm and manylm functions in the R package mvabund^38^, with statistical inferences from likelihood ratio tests using the anova function and with Monte Carlo resampling for abundance models and residual resampling for biomass models, with 1,000 iterations. Model assumptions (here and for and all mvabund models hereafter) were checked as per Wang et al.^40^ using mean–variance versus fitted plots (manyglm, manylm) and mean–variance relationship plots (manylm). Transformations were made where the mean–variance assumption was not met.

Benthic assemblages were characterized using photo-quadrats (50 cm × 50 cm) taken every meter (25 per transect) by a diver swimming at least 5 m behind the diver undertaking the fish counts. Three points were randomly overlaid on each image using CPCe (National Coral Reef Institute, v4.1), and the taxon or substratum underneath each point was identified, giving 75 points per transect. Each point was classified using the following eight categories: kelp, other macroalga (*Sargassum* and macroalgae), table coral, other live coral (all live coral besides table coral), turf and calcareous algae, dead coral (all dead corals), rocky reef and bare (bare rock and sand). The benthic community matrix was contrasted among habitat type (coral, kelp; fixed factors) and location (coral: Tei, Yokonami; kelp: Usa, Tanoura; fixed factors nested in habitat) using a multivariate linear model and the manylm function in R. Statistical inferences were carried out using likelihood ratio testing with the anova function and residual resampling with 1000 iterations.

### Feeding preference experiments

We set up the feeding experiments in shallow reef habitat (1–3 m), in patches dominated by a thin layer of epilithic algal matrix (EAM) cover sensu Wilson et al.^38^ within the coral-dominated locations (Tei and Yokonami). For each replicate assay, we tied individuals of the six seaweeds at 15 cm intervals to a 1 m rope that was weighed down at both ends. We aimed to keep initial biomass values as similar as possible between species within each replicate assay, however *Ecklonia cava* was always heavier than all other species and *Sargassum micracanthum* always lighter. The order in which the six seaweeds were attached was haphazard and varied between replicates. Each replicate assay included a paired control assay that was protected from herbivores by a 3 mm mesh cage (120 × 30 × 30 cm) to control for any biomass change not resulting from herbivory (e.g., handling losses and algal detachment resulting from water movement). At each location and time, we deployed between 8 and 15 replicate assays over a span of 2–3 days: a total of 9 assays were deployed at Tei in July and 15 in October, and 7 at Yokonami in July and 15 in October 2014. To maximize independence among replicates, we separated individual replicate assays by at least 5 m and positioned them in a different location each day. In October 2014, an additional set of feeding assays were performed using only *Ecklonia cava,* as this was the only species still remaining at that time of the year. The water temperature during the experimental period was about 26 °C in July and about 24 °C in October.

We deployed assays over intervals of 3 h, between 10:00 a.m. and 5:00 p.m. because herbivorous fishes are generally diurnal in the wild^39^. We pat dried the macroalgae to remove excess water and measured fresh weight to the nearest 0.1 g before and after deployment. For each species, we calculated the biomass lost by subtracting the proportion of alga lost from the caged controls (initial g − final g/ initial g) from the proportion of biomass lost from the uncaged treatments uncaged–caged;^40^.

Video recording was started immediately after the assay was deployed. We filmed the deployed feeding assays for 2–4 h (mean ± SE = 202 ± 0.7 min) each day to quantify the seaweed consumption patterns of individual fishes. On each filmed replicate, video footage was analysed. We counted the total number of bites taken by individual fish on either the experimental seaweed species tethered to the rope or on the EAM surrounding the assays. Forays where rapid consecutive bites by an individual fish took place without discernable pauses were conservatively classed as a single bite, as it was not possible to accurately measure the number of bites.

Biomass loss was contrasted among seaweeds of tropical/ temperate origin (fixed factor) and among algal species (fixed factor nested within tropical/ temperate origin) as well as between locations (fixed factor) using a linear model with manylm. Statistical inferences were carried out with likelihood ratio tests using residual resampling with 1,000 iterations.

The number of bites taken by the four most common herbivorous fish species observed (*Calotomus japonicus*, *Kyphosus vaigiensis*, *Naso unicornis* and *Siganus fuscescens*) was also contrasted among tropical/ temperate origin of algae, among algal species and between locations using negative binomial generalised linear models with manyglm. Using the anova function, statistical inferences were carried out with likelihood ratio tests using Monte Carlo resampling with 1000 iterations.

### Temporal patterns in kelp consumption

Consumption of the kelp *Ecklonia cava* in October 2014 when offered as a single-species assay appeared to be higher than in July, when *E. cava* was offered alongside other seaweed species (Fig. 2). To quantitatively determine whether kelp consumption varies throughout the year, additional feeding experiments were conducted monthly throughout the year of 2018 at Yokonami. Six *E. cava* kelp individuals were deployed each month (mean ± SE = 50.6 ± 2.6 g), three as treatment and three as control with herbivore exclusion cages (2 mm mesh, 20 × 20 × 20 cm), except October (n = 2 for treatment and control, respectively). The kelps used in the experiment were in good condition (not wilted), with few epiphytes in all months. Reproductive sori were observed on kelp blades from September to December at Usa, where the experimental kelps were collected. Each replicate assay was haphazardly placed at least 20 m apart from each other. An underwater video camera was deployed in each of the three treatment kelps, and the kelps and cameras were retrieved two hours after the start of the experiment. The percentage of kelp removed was calculated from the wet weight of the seaweed before and after the deployment. The results were compared between the treatments (treatment vs. control; fixed factor) and the sampling months (January–December; fixed factor) by two-way ANOVA using natural log transformed data. Model diagnostics (residuals plots and Durbin–Watson test) were run using the DHARMa package^41^. No temporal autocorrelation was detected.

### Seaweed nutritional traits

The nutritional traits (carbon, nitrogen and phenolics) of the six seaweeds were compared. Individual thalli of each species (n = 5) were freeze-dried and ground. Carbon and nitrogen content were analysed using a CHN Elemental Analyser (TruSpec^®^Micro Series, Michigan) at the Mark Wainwright Analytical Centre at UNSW Sydney. Phenolic content was analysed colorimetrically using the Folin-Ciocalteu’s method using gallic acid as a standard. Phenolics were extracted from ground seaweed (4–4.5 mg) using methanol–water (1 ml; 1:1). Samples were shaken at room temperature for five minutes at 30 Hz using a Tissuelyser (Qiagen, WestSussex, UK) and incubated for 24 h at 4 °C prior to colorimetric analysis using the Folin-Ciocalteu reagent and 20% sodium carbonate solution. Absorbance was read at 765 nm with a fluorescent platereader (SynergyTM, Vermont). Total phenols were expressed as gallic acid equivalents (GAE % dry weight).

The total nitrogen content, C:N ratio, and total phenols (log transformed) were contrasted between tropical and temperate seaweeds (fixed factor) and among algal species (fixed factor nested within tropical/ temperate) with linear models using manylm. We used likelihood ratio testing and residual resampling with 10,000 iterations for statistical inferences.

To test whether any of the measured nutritional traits (N, C:N ratio and phenolics) is related to consumption of seaweed species by the dominant fish species observed biting (*Calotomus japonicus*), we performed a multiple regression with the lm function in R. The total number of *C. japonicus* bites for each assay was the response variable with the scaled covariates of the mean nitrogen, C:N ratio, and phenolics of each seaweed species as the predictor variables.

## Results

### Characterisation of herbivorous fish and benthic communities

**Figure 1:**
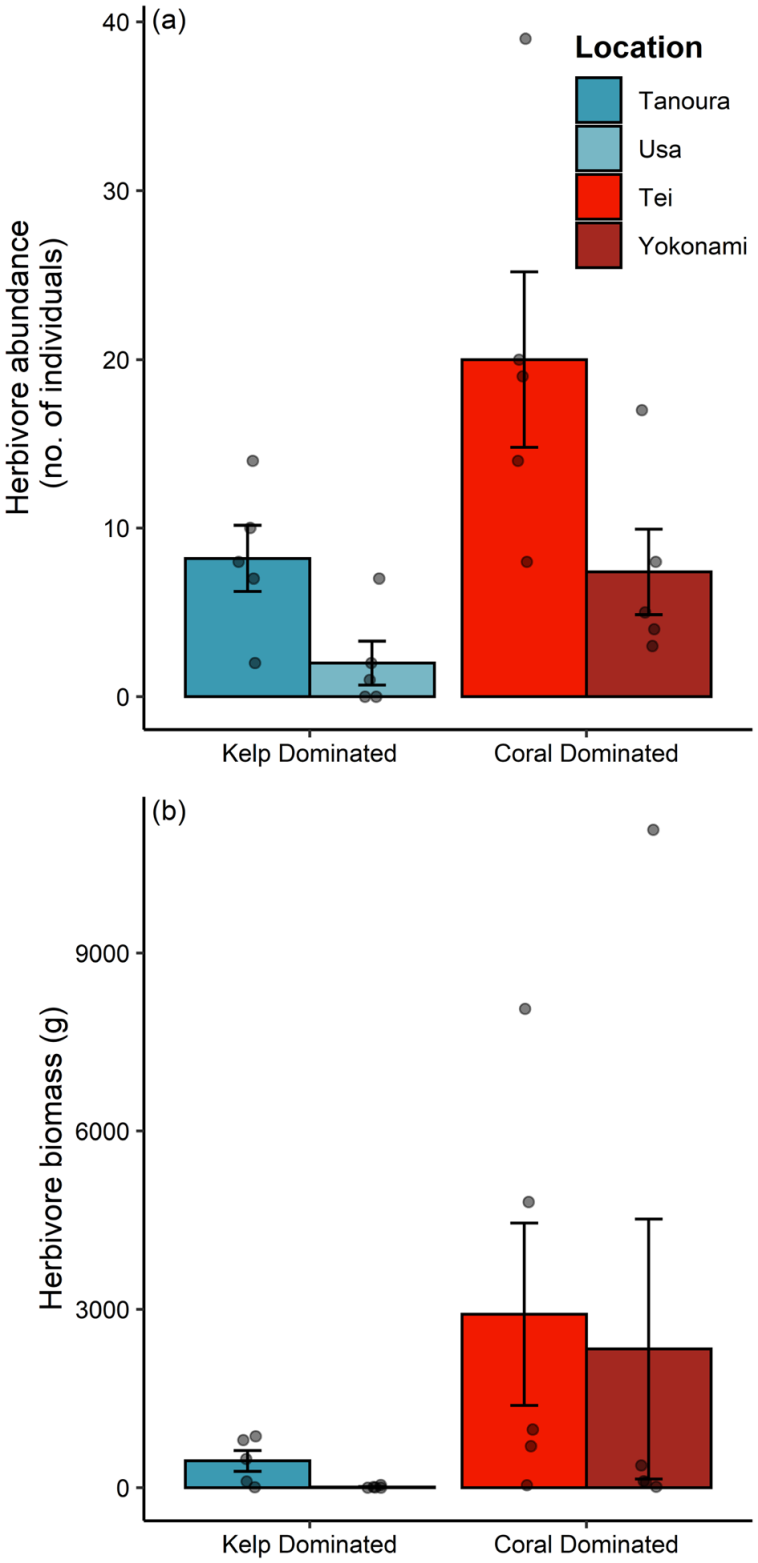
Total abundance of herbivorous fish (**a**), and total biomass of herbivorous fish (**b**) observed across kelp-dominated ‘temperate’ locations (in blue) and coral-dominated ‘tropicalised’ locations (in red). Bars are mean (± SE) and dots are the raw observations per transect.

Total herbivorous fish abundance differed between coral and kelp dominated habitats (df = 1, deviance = 5.41, *P* = 0.010) and among locations (df = 4, deviance = 11.16, *P* = 0.005; Fig. 1a). Total herbivorous fish biomass was higher at coral-dominated habitats in contrast to kelp habitats (F_1,18_ = 6.505, *P* = 0.015; Fig. 1b) and differed among locations (F_6,16_ = 1.163, *P* = 0.001026) driven by very low biomass of herbivores at Usa (Fig. 1b).

In the kelp habitat, *Prionurus scalprum* and *Girella punctata* dominated the herbivorous fish communities in terms of abundance (47% and 40% of the total herbivorous fish across the two kelp sites, respectively). However, their body sizes were mostly small (5–15 cm TL and 5–10 cm TL, respectively). In the tropicalized coral-dominated habitats, *P. scalprum* dominated the herbivorous fish communities in terms of abundance (71% of the total herbivorous fish across the two coral sites). The herbivorous fish community in the coral-dominated habitats was characterised by the high abundance of large size individuals of *P. scalprum* and *C. japonicus* (15–30 cm TL and 20–40 cm TL, respectively), as well as the presence of tropical surgeonfishes (*Acanthurus nigrofuscus*, *A. dussumieri*, *A. lineatus*, and *Naso unicornis*) and tropical parrotfish (*Scarus ghobban*).

The overall benthic community composition differed between kelp and coral dominated habitats (F_1,18_ = 7266.00, *P* = 0.002) and between locations (F_6,16_ = 46.00, *P* = 0.002). Temperate locations (Tanoura and Usa) were dominated by the kelp, *Ecklonia cava*, which had the highest percent cover at Usa and was completely absent from Tei and Yokonami (Fig. S2). Tropicalized locations (Tei and Yokonami) were dominated by live table coral, which was more common at Yokonami and completely absent from Tanoura and Usa (Fig. S2).

### Feeding preferences by herbivorous fish

We observed five herbivorous fish species taking a total of 7689 bites on the feeding trials: *Calotomus japonicus*, *Kyphosus vaigiensis*, *Naso unicornis*, *Siganus fuscescen*s and *Prionurus scalprum*. However, *P. scalprum* was only observed biting in four videos and was subsequently excluded from analyses. We observed the turtle *Caretta caretta* attempting to feed on *Ecklonia cava* once, but it failed to take a proper bite. There was no feeding observed in three replicate camera deployments in Tei; this data was excluded from the analysis as it provides no information on relative feeding preferences. A total of nine caged controls (three in Tei and six in Yokonami in July) were disturbed during the experiment allowing herbivores to consume the algae. The values given to these controls were the average initial and end biomass of undisturbed controls from that location. Final sample sizes in July were n = 14 for Tei, n = 13 for Yokonami; in October, n = 15 for Tei, n = 15 for Yokonami.

**Figure 2:**
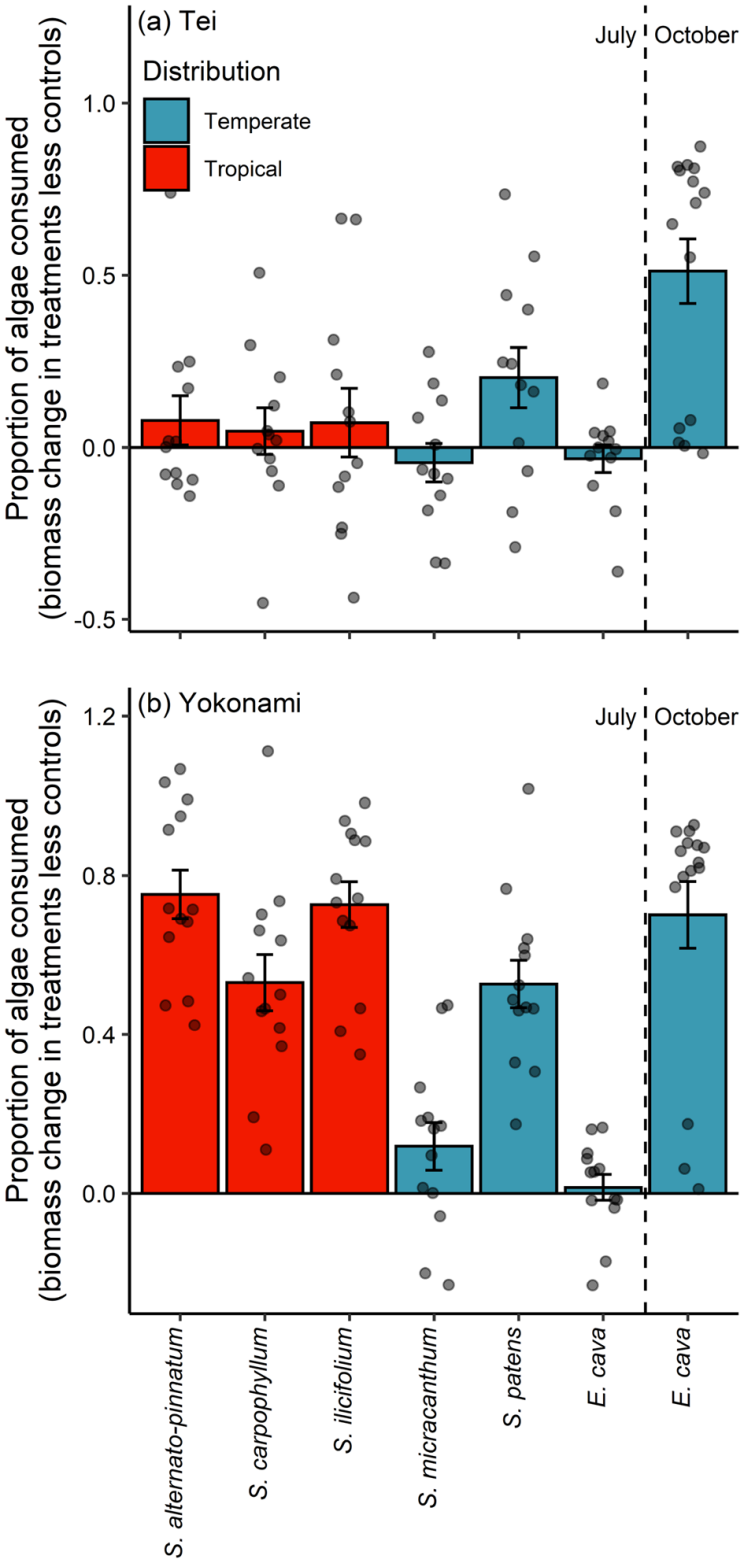
The proportional biomass loss from each seaweed species during feeding trials in (**a**) Tei and (**b**) Yokonami. Bars are the mean (± SE) algal biomass lost in treatments minus the algal biomass most in paired controls for each algal species and dots are the raw data.

Biomass loss was higher at Yokonami in contrast to Tei (F_1,178_ = 48.10, *P* = 0.002; Fig. 2) and was lower for temperate seaweeds (F_1,177_ = 4.49, *P* = 0.034). Algae biomass loss differed among seaweed species (F_6,171_ = 15.98, *P* < 0.001) but this was not consistent between locations (seaweed species and location interaction; F_6,166_ = 6.01, *P* < 0.001; Fig. 2).

**Figure 3:**
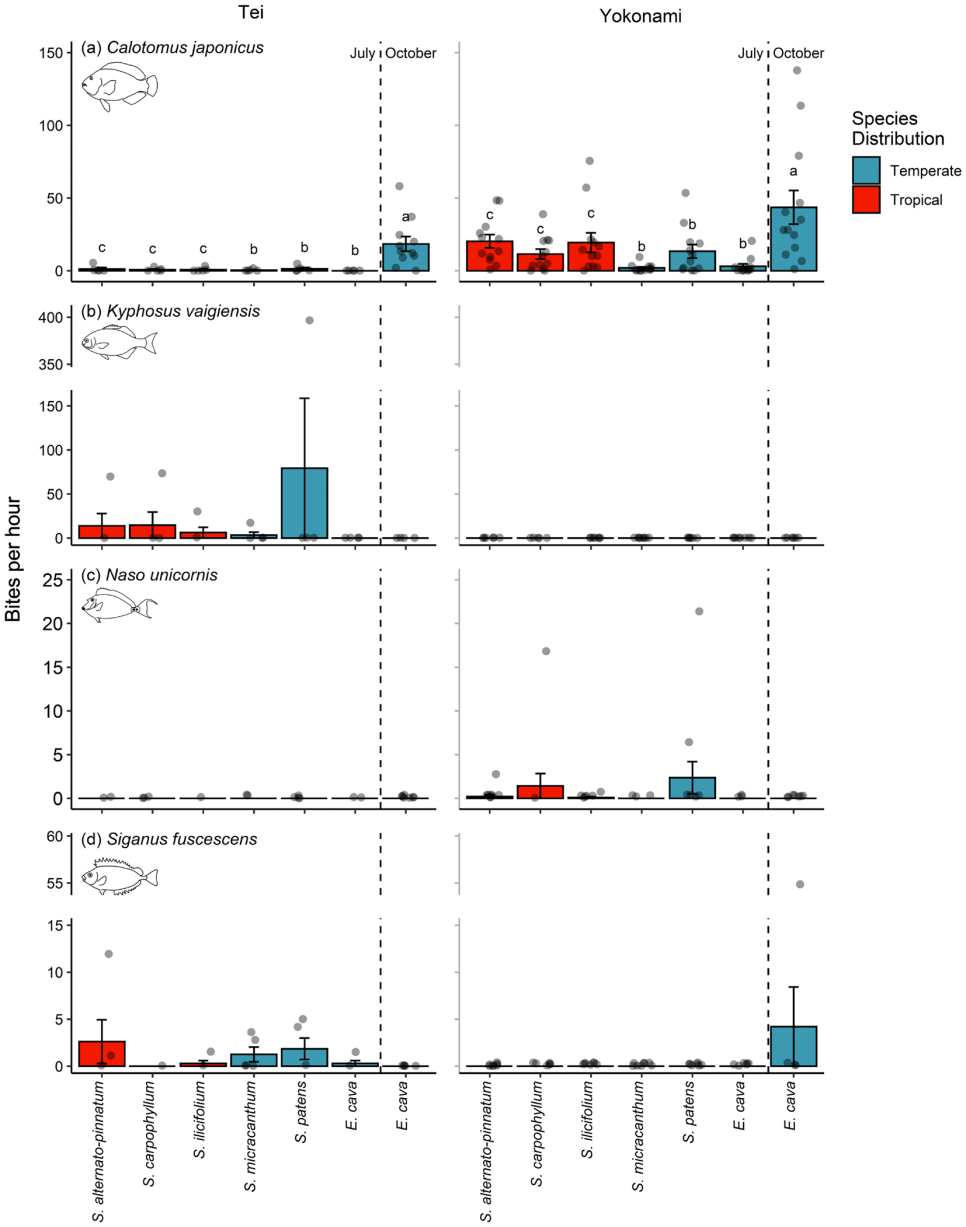
Number of bites taken per hour from each alga for the four (**a**) *Calotomus japonicus*, (**b**) *Kyphosus vaigiensis*, (**c**) *Naso unicornis*, (**d**) *Siganus fuscescens* on the two experimental sites: Tei (left side) and Yokonami (right). Bars are mean (± SE) bites per hour and dots are raw data. Bars sharing a letter did not differ among algae of the same distribution. Note truncated y-axis in (**b**) and (**d**).

The number of bites taken by *C. japonicus* was not influenced by whether an alga was of tropical or temperate origin (df = 1, deviance = 1.70, *P* = 0.193; Fig. 3a). However, *Calotomus japonicus* was observed feeding more often at Yokonami for four algae species (location and algal species interaction; df = 6, deviance = 14.28, *P* = 0.031; Fig. 3a). The kelp *E. cava* during October experienced the highest number of bites by *C. japonicus* at both locations (Fig. 3a). Bites by *K. vaigiensis* were not influenced by the tropical or temperate origin of seaweeds (df = 1, deviance = 0.02, *P* = 0.885; Fig. 3b). *K. vaigiensis* was observed feeding more often at Tei (df = 1, deviance = 14.40, *P* < 0.001; Fig. 3b), driven mostly by the large number of bites taken from *Sargassum patens* in one of the videos (interaction between location and species; df = 7, deviance = 0.001, *P* = 0.015; Fig. 3b). Bites by *N. unicornis* were also not influenced by the orgin (tropical or temperate) of seaweeds (df = 1, deviance = 0.0001, *P* = 0.998; Fig. 3c) and were marginally more frequent at Yokonami (df = 1, deviance = 4.00, *P* = 0.051; Fig. 3c). *S. fuscescens* also fed equally on tropical/ temperate seaweeds (df = 1, deviance = 1.89, *P* = 0.171; Fig. 3d), and fed more often at Tei, except for in October where more bites were taken from *E. cava* in Yokonami (location and species interaction; df = 6, deviance = 12.14, *P* = 0.002; Fig. 3d).

### Temporal patterns in kelp consumption

**Figure 4:**
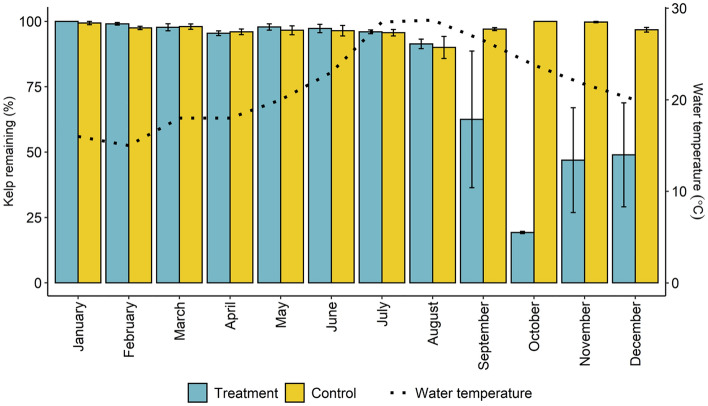
Monthly changes in the mean percentage remaining of the kelp biomass at Yokonami in 2018. The number of replicates is n = 3 for all months and for each treatment except October, where n = 2. Vertical bars indicate standard error. The broken line indicates the monthly change in seawater temperature during the experiment in each month at the study site.

We identified clear changes in kelp herbivory across the annual cycle in our assays, with the proportion of kelp biomass remaining after deployment being significantly lower in the herbivore-exposed treatments than in the controls, but only from September to December (treatment and month interaction; F_11,46_, *P* < 0.001; Fig. 4). Kelp herbivory was most intense in October (Fig. 4). Video analysis showed that all feeding was by *Calotomus japonicus*. In the months when there was no herbivory on kelp (January-July), some *C. japonicus* were also captured on video footage, but they were not observed feeding on the kelp bioassays.

### Seaweed nutritional traits

**Figure 5:**
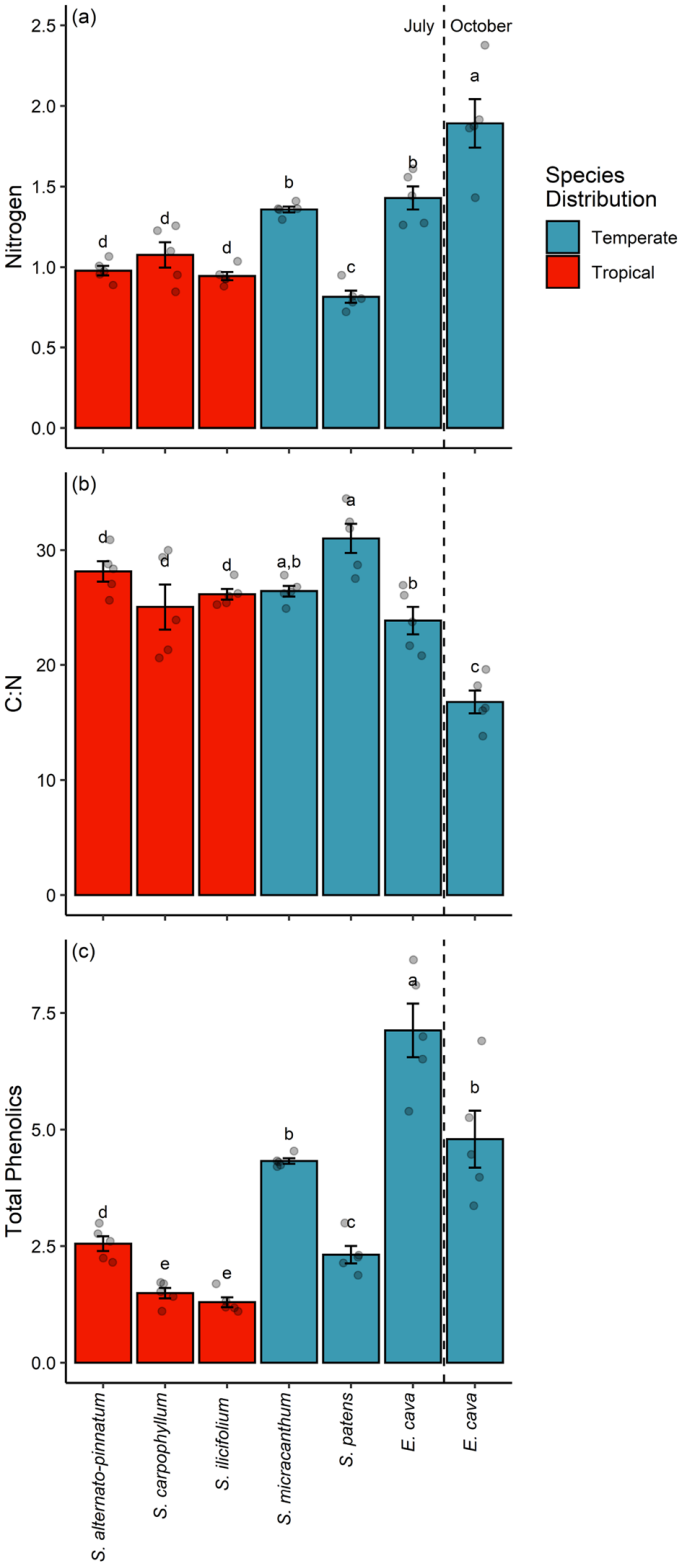
Nitrogen (**a**), C:N ratio (**b**) and total phenols (**c**) from each algal species in July and from *Ecklonia cava* in October. Bars that share a letter did not differ in Tukey’s post hoc tests contrasting algae from tropical and temperate distributions.

Nitrogen content differed between algae with tropical and algae with temperate distributions (F_1,33_ = 10.63, *P* = 0.003; Fig. 5a), with tropical algae generally displaying lower levels of nitrogen. Nitrogen content also differed among algal species (F_6,28_ = 18.02, *P* < 0.001; Fig. 5a). The C:N ratio differed among seaweed species (F_6,28_ = 13.66, *P* < 0.001) regardless of whether the species has a tropical or temperate distribution (F_1,33_ = 1.42, *P* = 0.236; Fig. 5b). The total phenolics differed among algal species (F_6,28_ = 23.36, *P* < 0.001) and were higher in temperate species in contrast to tropical species (F_1,33_ = 41.76, *P* < 0.001; Fig. 5c).

We found a significant negative relationship between the number of bites by *C. japonicus* and increasing nitrogen content (r^2^ = 0.099, F_1,98_ = 10.924, *P* = 0.001; Fig. S3a), no relationship with C:N ratio (r^2^ = 0.029, F_1,98_ = 1.040, *P* = 0.310; Fig. S3b), and phenolics (r^2^ = 0.079, F_1,98_ = 0.05, *P* = 0.831; Fig. S3c).

## Discussion

In this study, we found no evidence that temperate brown seaweeds are more palatable to herbivorous fish than tropical species, as originally hypothesised. Although nitrogen content was slightly higher in temperate species, none of the herbivorous fish observed feeding displayed a preference for temperate species, and overall tropical species declined in biomass at higher rates. Consumption of the declining kelp *Ecklonia cava* was low when this species was offered alongside tropical and temperate *Sargassum* species, which were generally preferred by all herbivorous fish. Kelp consumption, however, varied over a yearly cycle and was highest during the late summer and the autumn, when *Sargassum* species are absent due to natural senescence. High consumption rates on *E. cava* in the autumn coincide with the reproductive season for this kelp^42^, and may contribute to the long-term historical decline of this kelp in Tosa Bay^23^.

Kelp consumption was dominated by the parrotfish *Calomotus japonicus*, one of the most common endemic herbivorous fish in southern Japan. This parrotfish has also been identified as a key consumer of other kelp species in warming reefs elsewhere in Japan^43^. Since the highest rates of kelp consumption in the autumn coincide with the reproductive season of *E. cava*, it is possible that *C. japonicus* may be targeting reproductive tissue in plants. The herbivorous labrid *Odax pullus* is also known to target reproductive receptacles on brown algae^44^. However, although high consumption rates on *Ecklonia cava* in the autumn coincided with the highest nitrogen content recorded and nitrogen accumulation is associated with sorus formation^45,46^, we found an overall negative relationship between nitrogen content and *C. japonicus* consumption rates in our multiple choice assay.

Another possible explanation for the observed changes in *Ecklonia cava* consumption by *C. japonicus *may be linked to kelp surface-associated epibionts. Recently, Clements et al.^37^ hypothesized that browsing parrotfish target protein-rich epiphytes and microbes (e.g. cyanobacteria) on macrophytes. Some studies show that the surface microbiome of kelp changes seasonally and with ocean temperature^47^, and it is therefore possible that *C. japonic*us respond to those kind of changes. The broad preference by multiple herbivorous fish towards *Sargassum* could also be related to the epibionts. Indeed, several studies have suggested that herbivorous fish in the tropics known to consume Sargassum may be targeting epiphytes living on the seaweed rather than the seaweed itself^48,49^. The *Sargassum* species used in the feeding assays have a more complex structure at small scales (i.e. a greater number of small lateral thalli) than the kelp *Ecklonia cava*, which has a flatter, more laminar structure. The greater structural complexity of *Sargassum* species may create more suitable microniches for the accumulation of epiphytes and microbes, which may lead to greater consumption of *Sargasssum* by herbivorous fish.

Overall, seaweed biomass loss was slightly higher for tropical taxa. Tropical species also displayed lower levels of phenolic compounds, which are considered as deterrents for some herbivores^50^ although not all species are affected^51^. In this study, none of the individual herbivorous fish showed a preference for phenolic-poor tropical taxa. Other nutritional and structural components not measured here may also have influenced feeding preferences, as traits such as lipids and a broad range of secondary metabolites and structural materials have been previously linked to herbivore feeding patterns^52–54^.

Consumption of brown canopy-forming seaweeds was dominated by just four herbivorous fish. This finding is consistent with multiple studies from coral reefs, where a small number of species drive consumption patterns of seaweeds such as *Sargassum* in the Indo-Pacific^55,56^. Further, three of the species identified as the main consumers here, the drummer *Kyphosus vaigiensis*, the surgeonfish *Naso unicornis* and the rabbitfish *Siganus fuscescens*, are among the species most frequenly observed feeding on brown seaweeds in coral reefs^55,56^. *S. fuscescens*, and to a lesser degree *K. vaigiensis*, have also been identified as key consumers of temperate kelp in tropicalised regions of Australia where kelp is declining and the abundance of tropical and subtropical herbivores is increasing^11,57,58^. Further, two congeneric *Siganus* species morphologically similar to *S. fuscescens* are the main consumers responsible for overgrazing canopy forming seaweeds in the Mediterranean^12^, thus confirming the importance of a small number of taxa in driving climate-mediated phase shifts.

The tropicalization of coastal environments can occur heterogeneously in space and time within the same region^13^. In some locations, the changes may occur rapidly, while in others they may be slower. If herbivorous fishes are partly responsible for tropicalization, herbivores are expected to be fewer in the kelp-remaining locations than in the tropicalized locations. Our study supported this assumption. The water temperature in the locations where kelp remains (Usa and Tanoura) is about one degree Celsius cooler than in other areas throughout the year, probably because there is a river nearby in each location, which may provide suitable conditions for kelp survival and slow down the invasion rate and growth of herbivorous fish populations under ocean warming. The higher biomass of herbivores in tropicalised reefs was driven by the abundance of large browsing herbivores such as *Calotomus japonicus* and by large grazers such as the surgeonfish *Prionurus scalprum*. Although *P. scalprum* were generally not observed consuming large quantities of seaweeds, they were observed biting surrounding turf and epilithic algae, which may contain recruits of *Sargassum* and kelp. This species may thus play a role in keeping reefs in coral-domianted states by preventing the reestablishment of seaweeds through cropping^10,11^. A similar ecological role may also be played by other grazers/scrapers, such as tropical surgeonfishes (*Acanthurus* spp.) and parrotfish (*Scarus ghobban*) that occurred abundantly in coral-dominated locations. The increase in tropical herbivorous fishes in addition to native temperate herbivorous fishes is thought to increase the diversity of herbivory functions that promote the decline and prevention of the recovery of seaweed beds, as seen in Western Australia^59^. Since the loss of kelp beds in Tosa Bay is linked to high water temperature stress during the summer months^23^, our results suggest a scenario where high herbivory during the kelp reproductive and recruitment seasons exacerbate this decline.

To conclude, our findings suggest that the shift from temperate to tropical *Sargassum* species observed in Tosa Bay is probably not related to changes in fish herbivory, and may instead be caused by direct physiological effects from ocean warming on the seaweeds. In contrast, the dramatic historical decline in *Ecklonia cava* kelp forests observed in Tosa Bay^23^ is likely to be not only due to direct physiological effects on the kelp^27^, but may also be at least partly driven by high feeding pressure on adult kelp during times of the year when other seaweeds are not abundant and when the kelp itself is reproductive. This information contributes to our understanding of shifting trophic interactions in warming ecosystems and can inform coastal conservation measures such as seaweed restoration efforts in response to the decline of seaweed beds associated with ocean warming.

## Supplementary Information

Supplementary Figures.

## Data Availability

Data and code will be made available through Zenodo (upon publication). Direct requests for data from this study can be made to Adriana Vergés (a.verges@unsw.edu.au).
