# Supplementary material for: Differences in fish herbivory among tropical and temperate seaweeds and annual patterns in kelp consumption influence the tropicalisation of temperate reefs
Source: Sci Rep. 2022 Dec 8;12:21202. doi: 10.1038/s41598-022-24666-9 (PMC9731966; doi:10.1038/s41598-022-24666-9)
Supplement: Supplementary file 1 — Supplementary Figures. [file 41598_2022_24666_MOESM1_ESM.docx]

**Supplementary material**


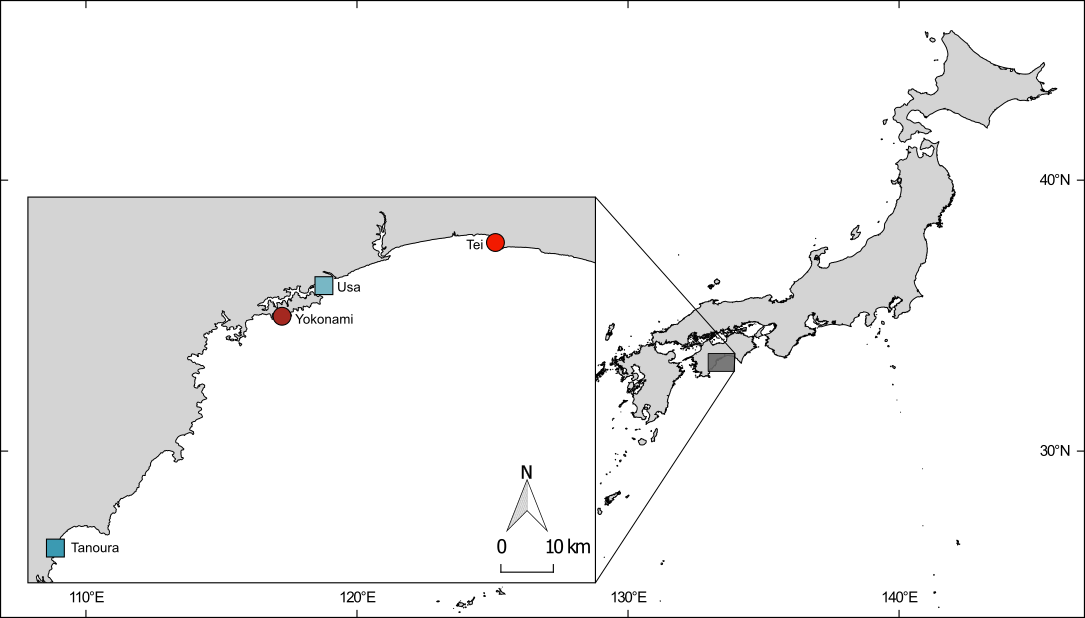


**Figure S1**. Map showing the four study sites in the Kochi Prefecture of southeastern Japan, with red circles representing the coral-dominated sites and blue squares representing the kelp-dominated sites. This map was generated using the ggmap package in R, accessible via: <https://cran.r-project.org/web/packages/ggmap/readme/README.html>


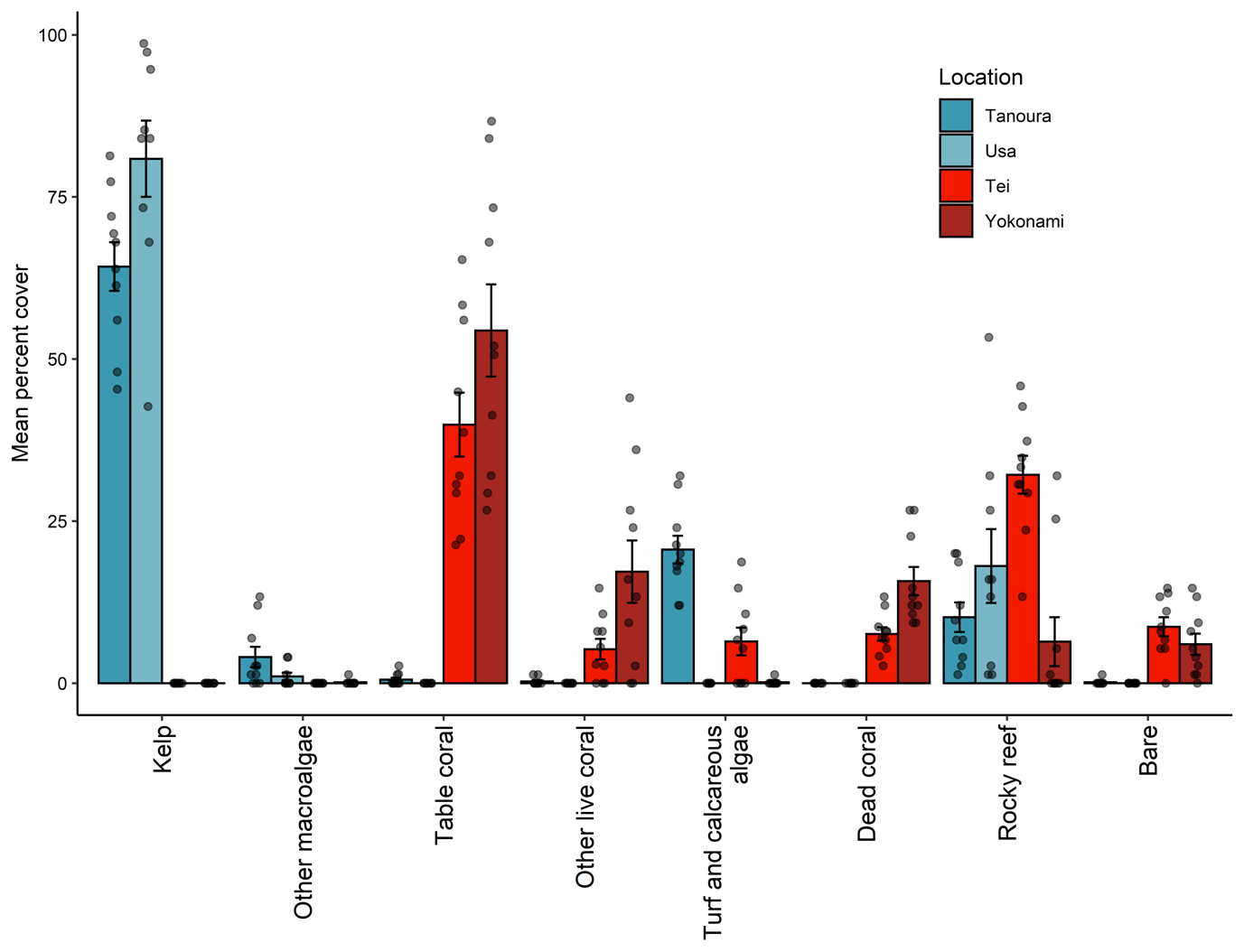


**Figure S2.** Percent cover recorded from benthic transects at each location. Bars are mean percent cover (± SE), dots are the raw data showing percent cover per transect. To help visualize the data, categories have been pooled in this plot but were analysed separately.

**
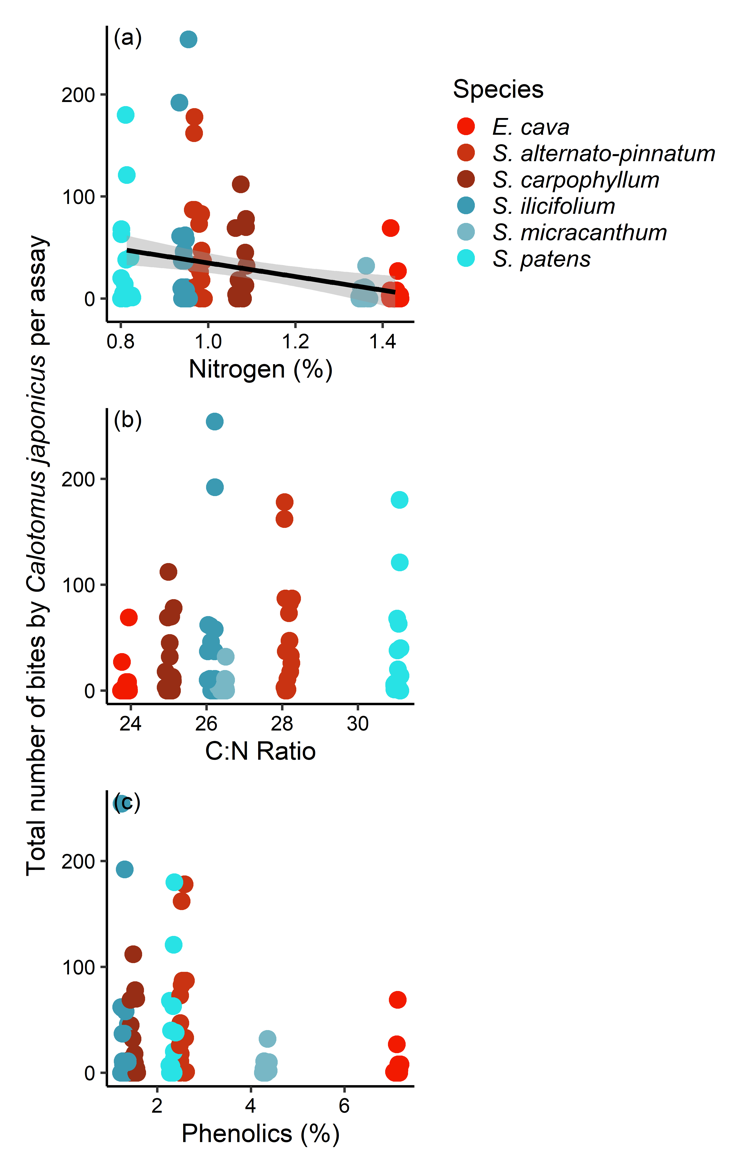
**

**Figure. S3.** Multiple regression showing the correlation between the total number of bites taken by *Calotomus japonicus* for each assay and the nutritional traits of the seaweeds consumed.
